# Genome evolution of *Acinetobacter baylyi* ADP1 during laboratory domestication: acquired mutations impact competence and metabolism

**DOI:** 10.1128/aem.00936-25

**Published:** 2025-08-19

**Authors:** Isaac Gifford, Meghna R. Vergis, Jeffrey E. Barrick

**Affiliations:** 1 Department of Molecular Biosciences, Center for Systems and Synthetic Biology, The University of Texas at Austin 196204, Austin, Texas, USA; Danmarks Tekniske Universitet The Novo Nordisk Foundation Center for Biosustainability, Kgs. Lyngby, Denmark

**Keywords:** laboratory evolution, domestication, natural competence, *Acinetobacter baylyi*, post-transcriptional regulation

## Abstract

The bacterium *Acinetobacter baylyi* is a model organism known for its extreme natural competence and metabolic versatility. It is capable of taking up environmental DNA at a high rate across all growth phases. The type strain ADP1 was created by random mutagenesis of a precursor strain, BD4, to prevent it from forming cell chains in culture. ADP1 has since been distributed between research groups over several decades and acquired subsequent mutations during this time. In this study, we compare the genome sequences of *A. baylyi* BD4 and its modern descendants to identify and understand the effects of mutations acquired and engineered during its domestication. We demonstrate that the ADP1 variants in use today differ in their competence, growth on different carbon sources, and autoaggregation. In addition, we link the global carbon storage regulator CsrA and a transposon insertion that removes its C-terminal domain specifically to changes in both overall competence and an almost complete loss of competence during the stationary phase. Reconstructing the history of ADP1 and the diversity that has evolved in the variants currently in use improves our understanding of the desirable properties of this experimentally and industrially important bacterium and suggests ways that its reliability can be improved through further genome engineering.

**IMPORTANCE:**

*Acinetobacter baylyi* ADP1 is a bacterial chassis of interest to microbiologists in academia and industry due to its extreme natural competence and wide metabolic range. Its ability to take up DNA from its environment makes it straightforward to efficiently edit its chromosome. We identify and characterize mutations that have been passed down to modern strains of ADP1 from the initial work in the 1960s, as well as subsequent mutations and genome edits separating strains in use by different research groups today. These mutations, including one in a global regulator (CsrA), have significant phenotypic consequences that have affected the reproducibility and consistency of experiments reported in the literature. We link a mutation in this global regulator to unexpected changes in natural competence. We also show that domesticated *A. baylyi* strains have impaired growth on a variety of carbon sources.

## INTRODUCTION

Microbes isolated from natural environments frequently find uses in laboratories as model systems for understanding biology or platforms for industrial applications. Many of these strains have been intentionally mutated to improve specific properties (1, 2). Even when strains have not been intentionally engineered, however, their genotypes do not remain static over time due to evolution (3–7). Because isolates of commonly used strains are also frequently shared between research groups, mutants with altered properties can also be unintentionally disseminated. Mutants may lose traits that are unnecessary in laboratory cultures, such as motility (8) or extraneous metabolic pathways (5, 7). These changes in phenotype are frequently the result of mutations in global regulators that control several genes with varied functions (3, 7). Growth of strains in lab cultures can also unintentionally result in the gain of phenotypes such as antibiotic resistance (4). This evolution can occur on short timescales, due to a single mutation taking over within a population after a few generations of use in the lab (3, 4), or on longer timescales as multiple mutations accumulate over years of routine use (2, 4).

The bacterium *Acinetobacter baylyi* ADP1 has been used in a broad range of applications in academic and industrial settings (9). ADP1 is highly naturally competent, able to bind and take up DNA in a non-sequence specific manner from the environment through the action of its competence pilus (10). It is known for the rare and, to date, unique trait of maintaining high competence in culture across different stages of normal growth (11, 12). This trait makes it straightforward to engineer its genome (13), as well as making it a model organism for studying the mechanism of natural competence (10) and its impact on genome evolution (14). ADP1 is also of interest for industrial applications, ranging from lignin valorization to bioremediation, because it can consume and synthesize a variety of relevant and desirable compounds (15–17).

The BD4 strain that would become ADP1 was originally isolated from soil based on its ability to grow on 2,3-butanediol (18). Subsequently, Elliot Juni and Alice Janik mutated the strain with UV to isolate a colony that did not form cell chains in culture and had a reduced capsule (1). This strain was designated BD413. Elliot Juni deposited these original strains, BD4 and BD413, with the American Type Culture Collection under the accession numbers ATCC 33304 and ATCC 33305, respectively. These strains were originally classified as *Acinetobacter calcoaceticus*, then as an unnamed *Acinetobacter* species, and finally as *A. baylyi* (19). Strain BD413 has subsequently been passed between labs over the last six decades. The ADP1 designation of a BD413 stock from Nick Ornston’s lab is the standard one used for this strain today (19, 20).

In this study, we investigate mutations that evolved in *A. baylyi* ADP1 over six decades of laboratory domestication. We identify mutations tied to the original UV mutagenesis and laboratory propagation that resulted in multiple descendants of BD413 that are all referred to in the literature as ADP1. These ADP1 variants and the transposon-free variant ADP1-ISx, engineered from one of them (21), bear marked genotypic and phenotypic differences that affect key *A. baylyi* features, including its competence and growth on a variety of carbon sources. These findings will aid in the design of future experiments utilizing this model organism and promote clarity and reproducibility between different research groups.

## RESULTS

### *A. baylyi* ADP1 lost a 130-kb plasmid during domestication

**Fig 1:**
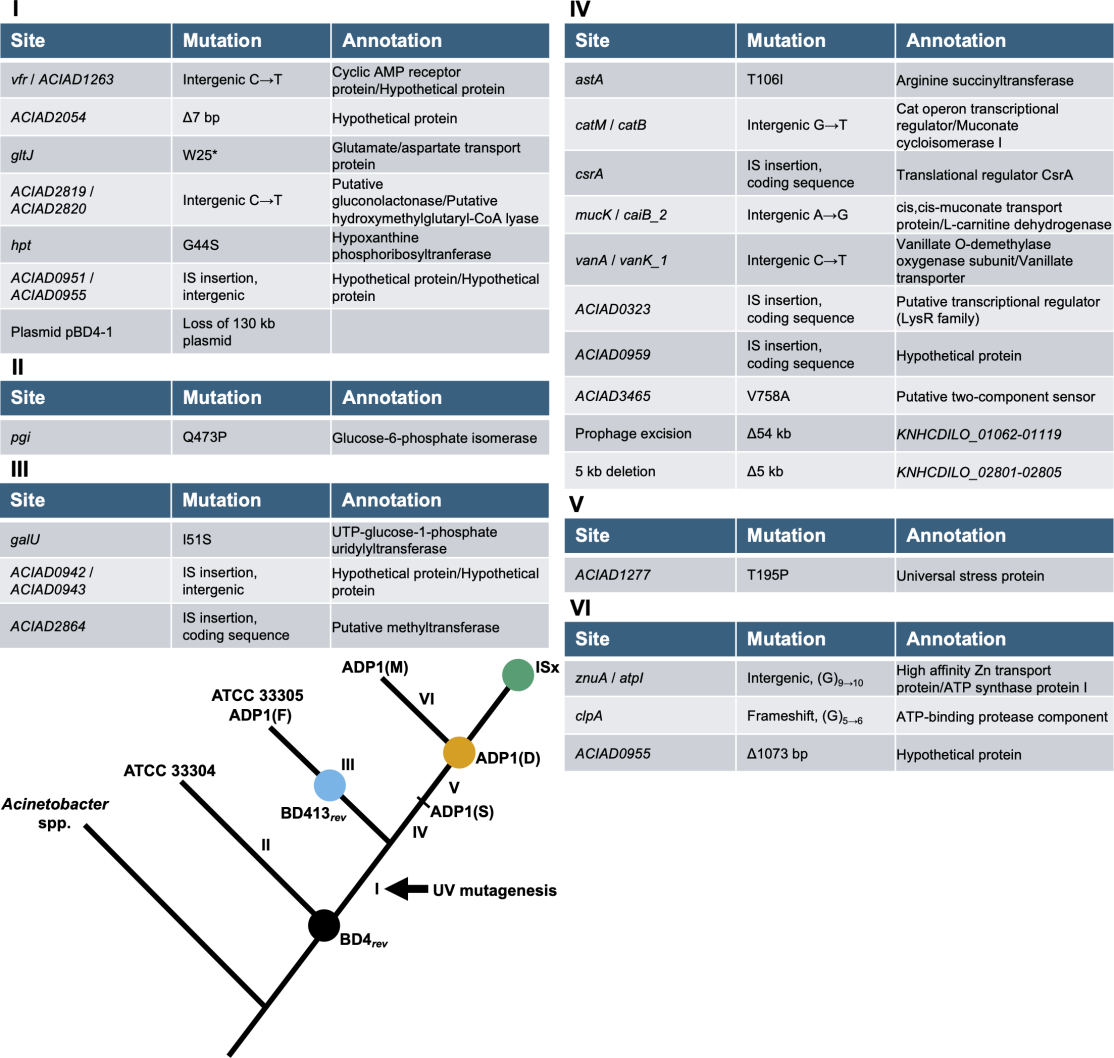
Maximum parsimony phylogeny of *A. baylyi* strains inferred from mutations that evolved during domestication. Strains were grouped by the presence or absence of mutations shown in Table S1. Branch labels correspond to sets of mutations that evolved along each branch. Targeted deletions of IS elements during the creation of strain ISx are described in (21). Circles indicate strains used in assays in this study.

We constructed a reference genome of *A. baylyi* strain ATCC 33304 (deposited as the ancestral strain BD4) from Nanopore reads and polished the assembly with Illumina reads (File S1). It consists of two circular contigs of 3,648,419 and 130,836 bp. These were annotated with Prokka (22) and ISEscan (23) to identify coding regions and insertion elements. Prokka identified 3,314 protein-coding genes, 76 tRNAs, and 7 sets of rRNA genes in the chromosomal assembly. ISEScan identified two complete IS elements in the chromosome: IS*3* and IS*256*. The smaller contig resembles a plasmid and was designated pBD4-1. This plasmid has 120 protein-coding genes and belongs to the R3-T7 group of *Acinetobacter* plasmids (24). It contains a p*dif* module (25) consisting of two p*dif* sites located approximately 5.3 kb apart. This p*dif* module encodes arsenate resistance proteins and a toxin/anti-toxin system, potentially tied to plasmid stability. The genes in this module could be exchanged with p*dif* modules in similar plasmids from other *Acinetobacter* spp. by site-specific recombination. pBD4-1 contains 12 complete IS elements that are predicted to be members of the IS*3*, IS*5*, IS*982*, and ISNCY families. The pBD4-1 plasmid was not present in any of the derived *A. baylyi* strains (Fig. 1; Table S1), suggesting it was lost early in domestication.

### Domesticated *A. baylyi* strains diverged into two major clades

Illumina sequencing reads from six domesticated *A. baylyi* strains (Table 1) were compared against the reference genome assembled for ATCC 33304. A point mutation in *pgi* was predicted in all the domesticated strains relative to the ATCC 33304 genome. However, our subsequent analysis demonstrated that this reflects a mutation that evolved in the ATCC 33304 strain and was not present in the original BD4 ancestor (see “Domestication of ADP1 altered cell morphology,” below) rather than a mutation arising in the domesticated lineage. Extant strains descended from *A. baylyi* BD4 differed from ATCC 33304 by as many as 21 mutations (Fig. 1). We inferred a maximum parsimony phylogenetic tree from the presence and absence of mutations in each genome. Seven mutations were present in all domesticated strains (Fig. 1, group I). Presumably, these occurred during or soon after mutagenesis by Juni and Janik. The domesticated strains then diverged into two groups depending on the presence or absence of either two specific IS insertions (Fig. 1, group III) or a set of 10 other mutations (Fig. 1, group IV). The first clade includes ATCC 33305, deposited at ATCC by Juni. It likely represents minimal adaptation to laboratory culture since the original mutagenesis of BD4. This strain was identical to the one used by the Friedman Lab (26), designated ADP1(F) here. The second clade is composed of three other strains used by present-day research groups, and thus the 10 additional mutations they share arose during routine use before a stock was distributed to these labs. The ADP1(D) and ADP1(M) (14) strains share a point mutation in a universal stress protein (Fig. 1, group V) not found in the ADP1(S) (27) strain. The ADP1(M) strain also acquired an additional three mutations not found in the other two strains (Fig. 1, group VI).

**TABLE 1:** *A. baylyi* strains used in this study

ID	Strain	Source lab	Reference
ATCC 33304	BD4	ATCC	This study
BD4*_rev_*	BD4	NA^*a*^	This study
ATCC 33305	BD413 (ADP1)	ATCC	This study
BD413*_rev_*	BD413 (ADP1)	NA	This study
ADP1(D)	ADP1	de Crécy-Lagard	Renda et al. (28)
ADP1(F)	ADP1	Friedman	Meroz et al. (26)
ADP1(M)	ADP1	McDonald	Sezmis et al. (14)
ADP1(S)	ADP1	Santala	Luo et al. (27)
ADP1-ISx	ISx	Barrick	Suarez et al. (21)

^*a*^ NA, not applicable.

### Domestication of ADP1 altered cell morphology

**Fig 2:**
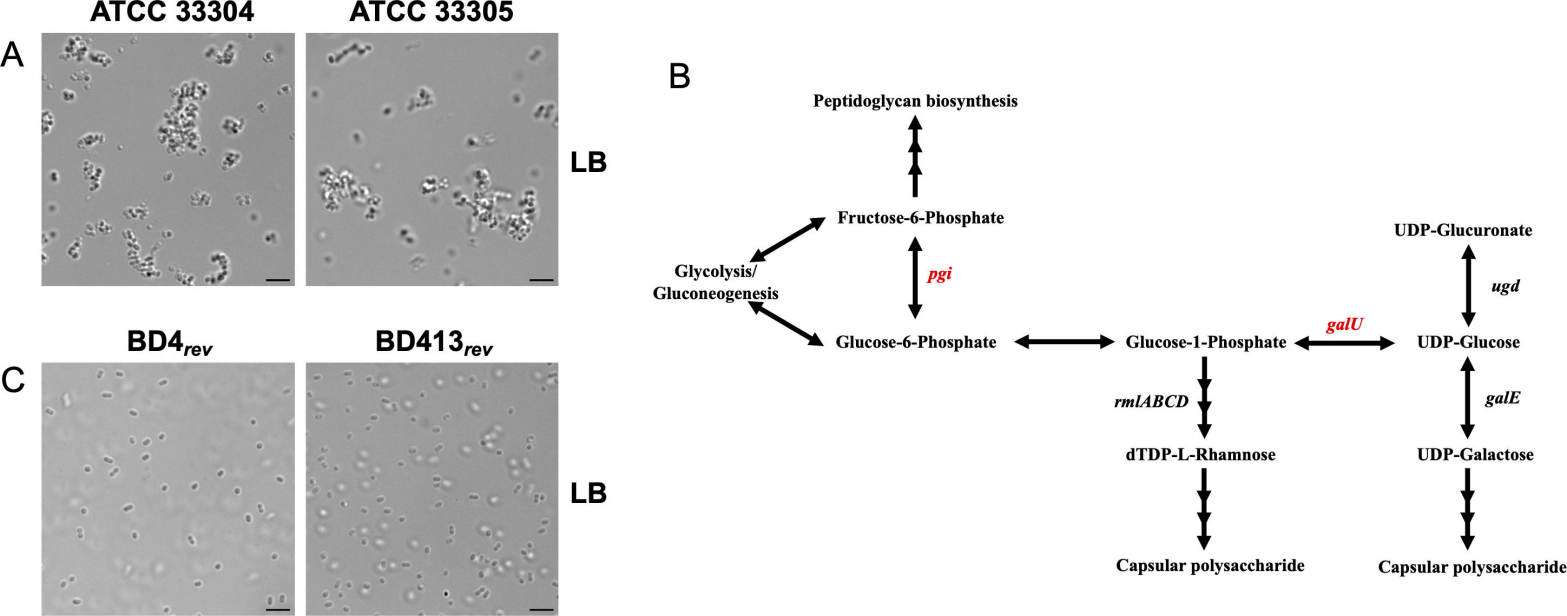
Mutations leading to aggregation in *A. baylyi* strains. (**A**) Microscope images of strains ATCC 33304 and 33305 grown in LB medium. (**B**) Metabolic pathways showing conversion of glucose to capsular polysaccharides. Genes mutated in ATCC 33304 and 33305 are shown. Pathways adapted from KEGG (29). (**C**) Reverting *pgi* or *galU* mutations in ATCC 33304 and 33305, respectively, produced single cells in LB. Scale bars represent 5 µM.

**Fig 3:**
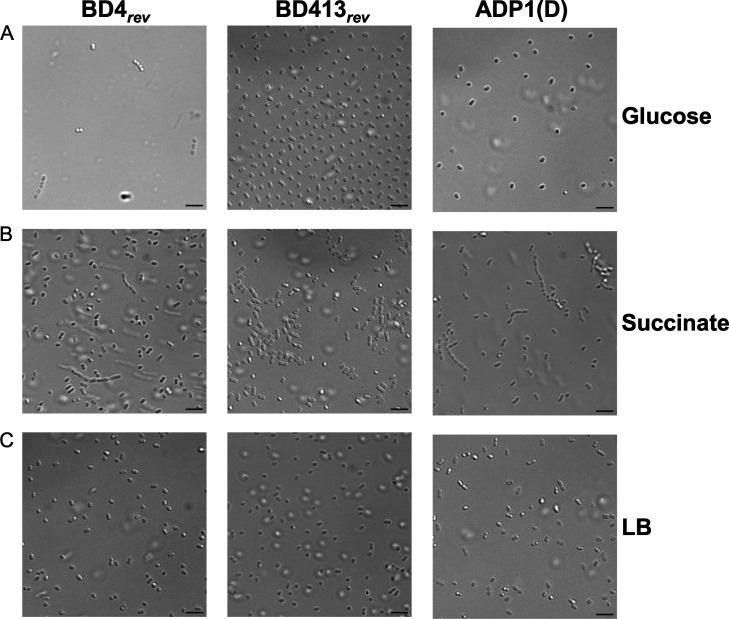
*A. baylyi* strains form cell chains in minimal media. (**A**) Domestication in BD413*_rev_* and ADP1(D) prevented the formation of cell chains in S2 medium with glucose. (**B**) Cell chains formed for all strains when succinate was used as a carbon source in S2 medium. (**C**) All strains, including BD4*_rev_*, grew unicellularly in LB medium. The image of BD413*_rev_* in LB is taken from the same field of view as in Fig. 2C. Scale bars are 5 µM.

Strains ATCC 33304 and 33305 grew in large aggregates in LB medium (Fig. 2A), whereas ADP1(D) and ISx grew as single cells. ATCC 33304 and 33305 each contained a mutation in a gene related to capsule biosynthesis: either *pgi* or *galU*, respectively (Fig. 1, groups II and III, and Fig. 2B). We reverted these mutations to create strains BD4*_rev_* and BD413*_rev_* (Fig. 2C), which are expected to be genetically identical or nearly so to the original BD4 and BD413 strains (1). As BD4 did not form aggregates in Juni and Janik’s original paper (1), this suggests that the *pgi* mutation arose later, as depicted in Fig. 1. Both BD4*_rev_* and BD413*_rev_* grew as single cells in LB medium and in the S2 minimal glucose medium used by Juni and Janik (1) (Fig. 3A), consistent with the results of their original mutagenesis. However, BD4*_rev_*, BD413*_rev_*, and ADP1(D) all grew as cell chains when succinate, commonly used as a carbon source for ADP1, was substituted for glucose (Fig. 3B). All three strains, including BD4*_rev_*, grew as single cells in LB medium (Fig. 3C).

### Mutation of *csrA* during domestication of ADP1 affected competence

**Fig 4:**
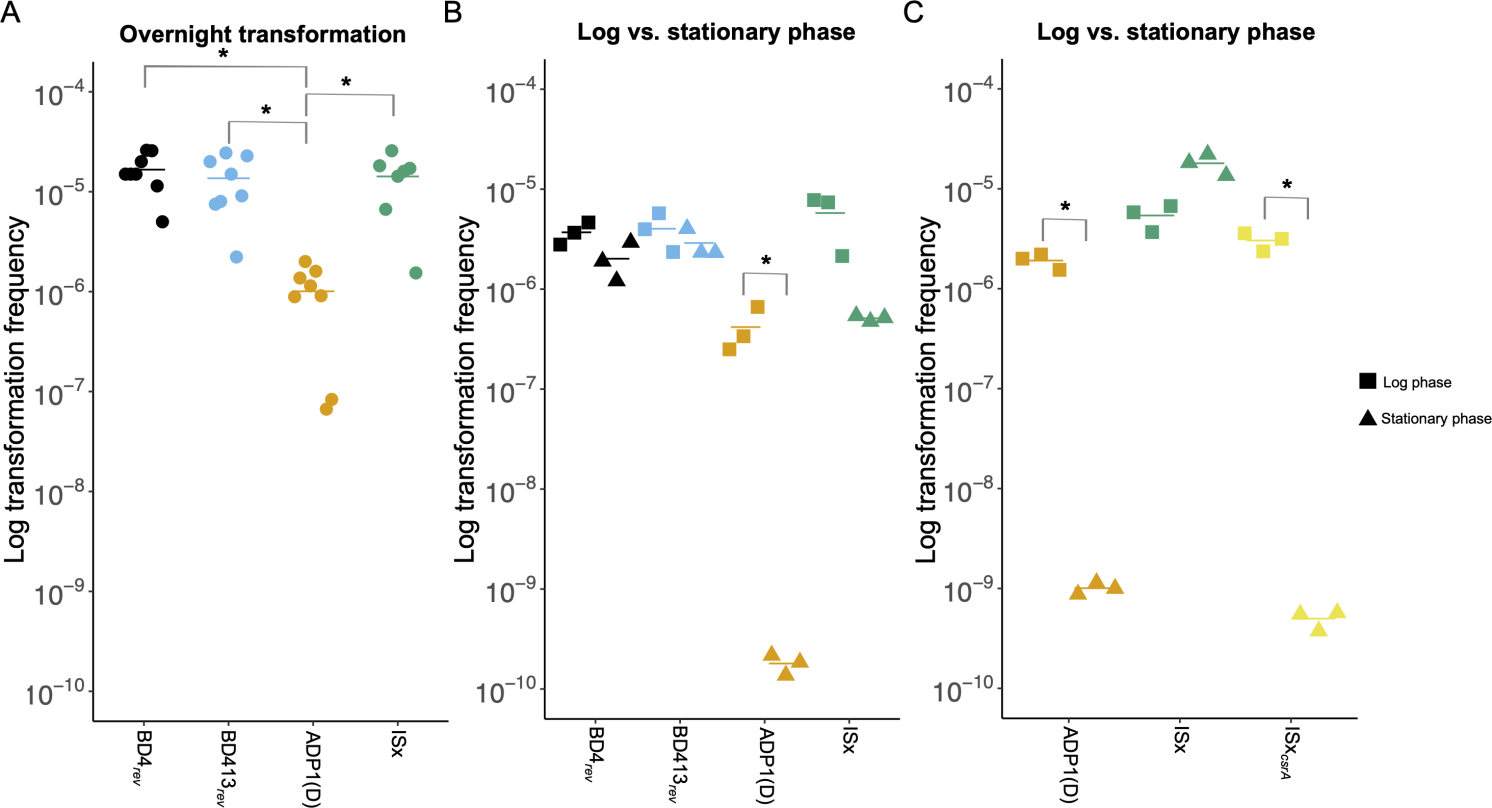
Transformation efficiency varies between domesticated *A. baylyi* strains. (**A**) Transformation frequencies of BD4_rev_ (black), BD413_rev_ (blue), ADP1(D) (orange), and ISx (green) strains incubated overnight with genomic DNA carrying a *specR* resistance gene. (**B**) Transformation frequencies of the same strains during log (squares) and stationary (triangles) phases. At each time point, each strain was incubated with transforming DNA for 30 min, then the remaining free DNA was digested by DNase I before plating to count transformants. (**C**) Transformation frequencies during log (squares) and stationary (triangles) phases for ADP1 and ISx compared to an ISx mutant with a truncated *csrA* gene (yellow). *P < 0.05 for Welch’s *t*-test.

Previous reports in the literature have differed on the competence of BD4 (1) and BD413 (11), particularly in their competence during stationary phase. To investigate whether these differences were caused by domestication, we performed competence assays on BD4*_rev_*, BD413*_rev_*, ADP1(D), and ISx. The domesticated strain ADP1(D) was significantly less competent than BD4*_rev_*, BD413*_rev_*, and ISx (pairwise Welch’s *t*-test, adj. *P* = 0.0025, 0.019, and 0.027, respectively). About 15-fold fewer ADP1(D) cells incorporated an antibiotic resistance marker with flanking homology to the chromosome in an overnight transformation assay (Fig. 4A). Competence in the other strains did not vary significantly. We sought to investigate whether the difference in competence in ADP1(D) was caused by changes in competence during different growth phases (Fig. 4B). BD4*_rev_* and BD413*_rev_* maintained high levels of competence in both log (3 h post-inoculation) and stationary (24 h post-inoculation) phases. ADP1(D), however, had >2,000-fold reduced competence during stationary phase. These results suggest that the reduced competence of ADP1(D) is due to a transposon insertion that truncated the global regulator *csrA* during domestication, a mutation that is only found in strains of one lineage of domesticated ADP1 and was removed during the construction of ISx (Fig. 1). A strain of ISx with the truncation recreated exhibited a similar >1,000-fold decrease in competence during stationary phase (Fig. 4C).

### Domestication of ADP1 impacted growth across different carbon sources

**Fig 5:**
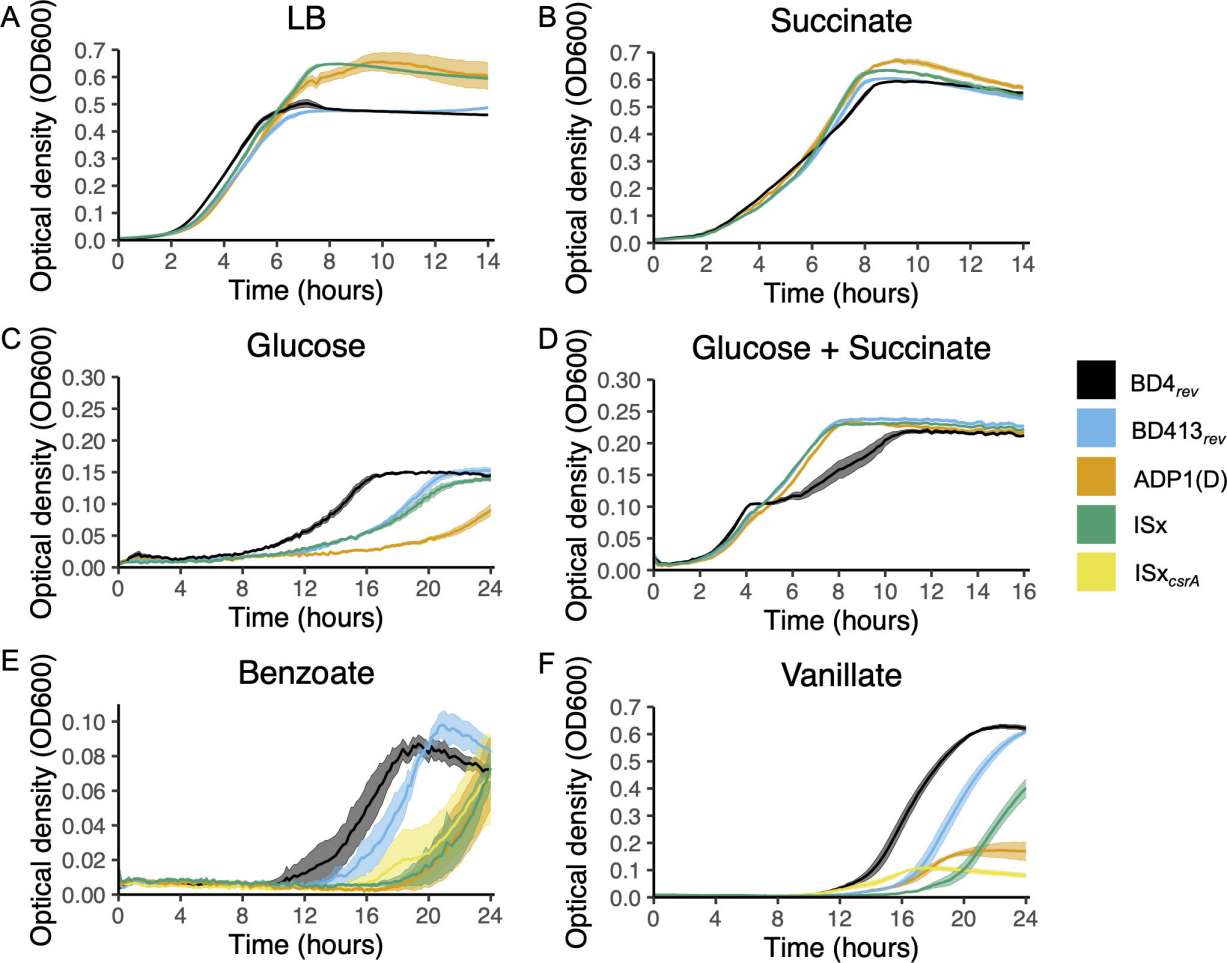
Domestication impacted growth and catabolite repression in *A. baylyi*. Growth curves of *A. baylyi* strains in (**A**) LB medium and in S2 media with (**B**) 25 mM succinate, (**C**) 3 mM glucose, (**D**) 3 mM glucose and 2.5 mM succinate, (**E**) 2 mM benzoate, or (**F**) 20 mM vanillate. Lines represent the average OD_600_ of six replicates each and error bars depict standard error. The strains tested were BD4*_rev_* (black), BD413*_rev_* (blue), ADP1(D) (orange), and ISx (green). E and F include an ISx mutant with the *csrA* truncation from ADP1(D) (yellow).

We investigated the growth of domesticated *A. baylyi* strains on different carbon sources to determine whether strain choice impacts its role as a model system for the degradation of aromatic compounds or as a chassis for metabolic engineering. When grown in LB medium, ADP1(D) and ISx grew to a substantially higher cell density than BD4*_rev_* and BD413*_rev_*, but did not show substantially higher growth rates (Fig. 5A). Further experiments were carried out in modified S2 media as described by Juni and Janik (1), but with other carbon sources substituted for glucose. In the presence of glucose, BD4*_rev_* reached its maximum density substantially faster than the domesticated strains, with ADP1(D) lagging further behind BD413*_rev_* and ISx, whereas in the presence of succinate (20 mM), there was no noticeable difference between the four strains (Fig. 5B and C). The lag observed in the domesticated strains when grown on glucose was rescued by adding a small amount of succinate to the media (2 mM, 1/10^th^ the normal concentration) (Fig. 5D). However, BD4*_rev_* displayed a two-step growth curve typical of catabolite repression in the mixed media, whereas the other strains showed only a single log phase. When grown on the aromatic compound benzoate (2 mM), the laboratory domesticated strain ADP1(D) and ISx displayed a longer lag phase than BD4*_rev_* or BD413*_rev_* (Fig. 5E). BD413*_rev_*, ADP1(D), and ISx also showed longer lag phases compared to BD4*_rev_* on vanillate (20 mM), and ADP1(D) grew to a particularly low optical density (Fig. 5F). Truncating *csrA*, mimicking the mutation in domesticated ADP1, reconstituted this phenotype in ISx.

### Adaptation of BD4_*rev*_ to laboratory culture

To investigate which mutations that evolved in ADP1 were likely caused by adaptation to laboratory culture, we “replayed” its domestication by evolving a BD4*_rev_* strain in *A. baylyi* minimal succinate (ABMS) medium. At the end of 1 month of daily serial transfers (~300 bacterial generations), we sequenced the genomes of endpoint clonal isolates, one each from 11 separate populations. We found that a base substitution leading to a K107E mutation in *ACIAD3105*, which encodes a DUF4124 domain-containing protein, had occurred in our ancestor stock before the start of the evolution experiment. While its appearance is further evidence of how rapidly new mutations can arise during laboratory culture, our focus was on investigating convergent evolution in ADP1 strains, so we did not further analyze its effects.

**Fig 6:**
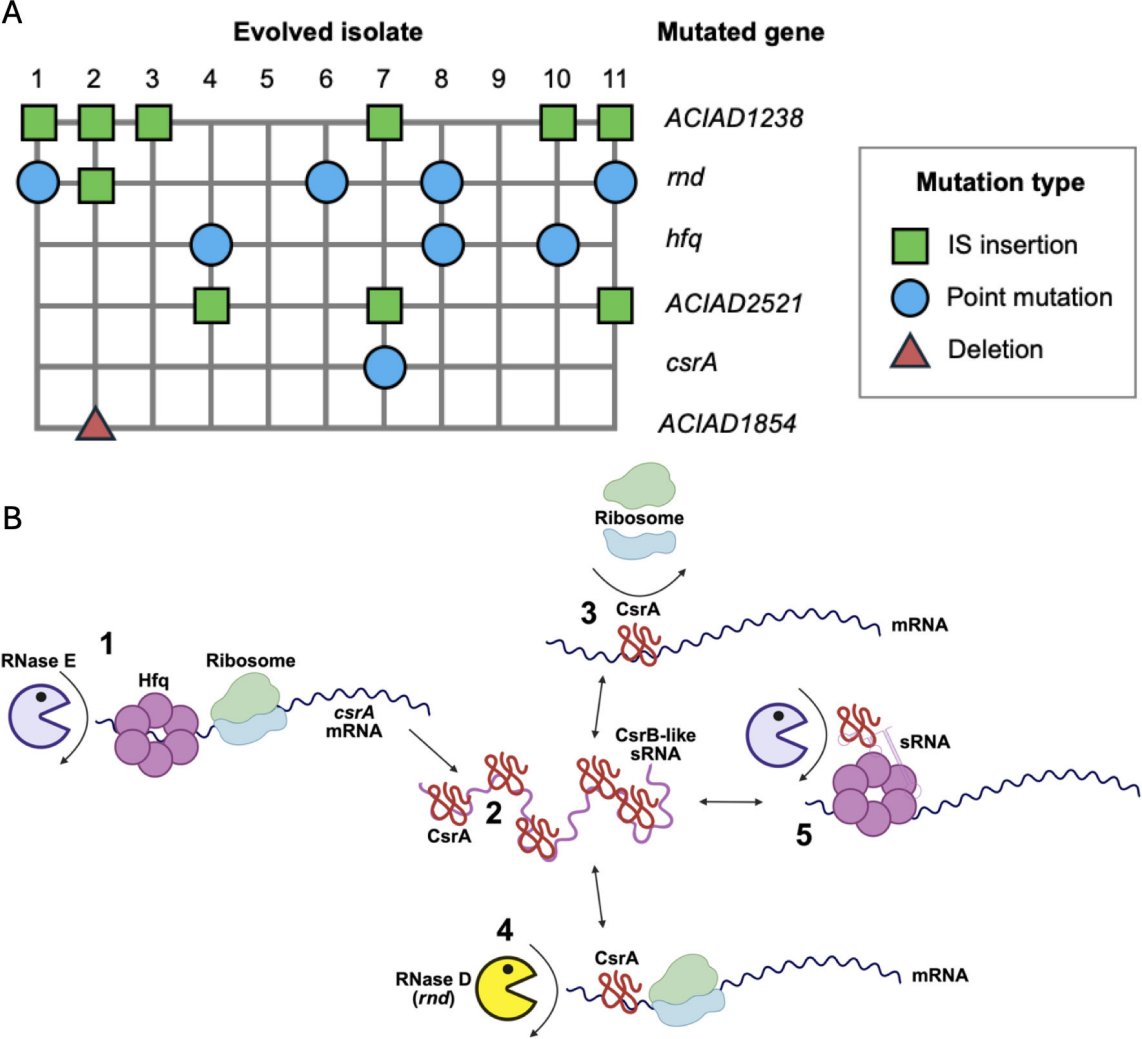
(**A**) Mutations evolved in BD4*_rev_* (MRV001) during 1 month of evolution in ABMS medium. Full details are provided in Table S1. (**B**) Potential post-transcriptional regulatory network inferred in *A. baylyi* from interactions between CsrA, Hfq, and RNase D. (1) Hfq protects *csrA* mRNA from degradation by RNase E (30). (2) CsrA proteins are bound by a CsrB-like small RNA (31). (3) CsrA binds to Shine-Dalgarno sites, preventing ribosome assembly on mRNAs (32). (4) CsrA protects mRNAs from degradation by RNase D (*rnd*). (5) CsrA protects small regulatory RNAs from degradation by RNases (33). Panel B created with Biorender.

Each endpoint isolate independently evolved between zero and three new mutations (Fig. 6A; Table S1). The predominant gene mutated was *ACIAD1238*, a stress response regulator. It experienced IS*982* element insertions in six isolates at four different positions between the −10 and −21 positions upstream of its start codon. The second most commonly mutated gene was ribonuclease D (*rnd*), which was disrupted in five endpoint isolates. Mutations in *rnd* included one out-of-frame insertion, one nonsense mutation, two missense mutations, and an IS*3* element insertion. One isolate had a missense mutation in the coding sequence of *hfq*, an RNA chaperone. Two isolates had a base substitution, either at 88 bp or 90 bp upstream of *hfq*. One isolate had a substitution at 7 bp upstream of *csrA*. Overall, 8 out of 11 isolates had mutations in *rnd*, *hfq*, and/or *csrA*. These mutations likely have convergent effects on a shared post-transcriptional regulatory network (Fig. 6B).

## DISCUSSION

### Mutations in capsule biosynthesis lead to autoaggregation in *A. baylyi* cultures

Through comparative genomics, we identified a set of mutations linked to the original mutagenesis that created *A. baylyi* strain BD413. However, these mutations only appear to have produced the desired phenotype (unicellular growth) in the original S2 glucose medium: cell chains were still observed when glucose was substituted for other carbon sources. Bacterial capsules can cause the formation of cell chains (34, 35); thus, it is likely that shrinking the capsule resulted in unicellular growth. These phenotypes are presumably tied to one or a few of the mutations we have linked to the original mutagenesis; however, growing the strains in different carbon sources restored the cell chains, potentially because different metabolic pathways or regulation restored full synthesis of the capsule.

In this study, we also demonstrated that some extant *A. baylyi* strains (ATCC 33304 and 33305) have acquired mutations causing autoaggregation. The capsule biosynthesis pathway in these strains has acquired mutations similar to those that have caused autoaggregation in previous *A. baylyi* evolution experiments (36, 37). As bacterial capsules can block autoaggregation by masking cell surface proteins such as adhesins (38), it is likely that the complete loss of the capsule, caused by mutations during routine culturing, exposes these proteins and allows them to interact. Future work could further promote *A. baylyi*’s ease of use by engineering a permanent solution to the issues of cell chains and autoaggregation through targeted deletions of capsule biosynthesis and cell surface adhesion proteins. This solution would have the added benefit of rerouting metabolic flux away from capsule biosynthesis, which may improve product yields (39).

### Truncation of *csrA* decreases competence of domesticated ADP1

We found that genetic diversity existed among several *A. baylyi* strains designated as ADP1. Divergence was especially notable between the strain deposited at ATCC (ATCC 33305) and those from different research groups. ATCC 33305 has undergone minimal evolution since the original mutagenesis performed by Juni and Janik (1). Strains in the other major clade were all obtained from academic labs that inherited their strains from other research groups. These strains have undergone further domestication and adaptation to laboratory culture over time. Strains in this clade evolved a shared set of mutations. Among these is the truncation of *csrA* (36), which led to a 10-fold decrease in transformation efficiency and an even larger decrease during stationary phase. Reversion of this mutation explains the increase in competence reported in the engineered ISx strain (21).

Reports on competence in *A. baylyi* have varied, particularly regarding its competence during different stages of growth. Juni and Janik originally described BD4 as a strain that was maximally competent at the onset of stationary phase, with competence decreasing during log phase and stationary phase (1). Subsequent work on BD413 described it as highly competent at all growth stages, including log phase (11, 12). We did not observe differences in competence between BD4*_rev_* and BD413*_rev_* or between log and stationary phase for either strain, supporting high competence across growth phases for *A. baylyi* strains with intact, ancestral *csrA* genes.

### *A. baylyi* adapts to laboratory culture through mutations in global post-transcriptional regulators

Previously, we reported that the strain ISx, created by deleting all insertion sequences from ADP1(D), frequently adapted in evolution experiments through mutations in *rnd* (RNase D), *hfq*, and *csrA* (36), whereas ADP1(D) (with its truncated *csrA* gene) does not (37). CsrA and Hfq are post-transcriptional regulators that affect the translation and stability of mRNAs. RNase D has also been shown in *Myxococcus xanthus* to regulate its lifestyle by degrading a specific transcript (40). It is likely that interactions between these proteins form a post-transcriptional regulatory network in *A. baylyi* (Fig. 6B). Here, we found that these genes also mutated in BD4*_rev_* during an evolution experiment. This consistent result suggests that truncation of *csrA* evolved from general adaptation to laboratory culture during domestication, rather than in response to the mutations introduced by UV mutagenesis in the history of ADP1.

CsrA is canonically involved in the regulation of carbon storage and catabolism (41), thus its mutation in ADP1 strains may be linked to modification of these pathways in laboratory culture. ADP1 strains often evolve reduced competence in laboratory experiments (37, 42), which has been ascribed to multiple factors, including gene expression burden from synthesizing the competence machinery, a fitness cost incurred from taking up homologous genomic DNA (42), and the emergence of a prophage that is thought to utilize its competence pilus as an attachment site (28). It is possible that truncation of *csrA* was selected for during domestication for the reduction in competence or for its effect on multiple processes.

In other bacteria mutations in global transcriptional regulators that respond to starvation and stress, such as RpoS in *E. coli* (3, 43) and *Salmonella enterica* (7), are common during laboratory domestication. Frequent mutations affecting post-transcriptional regulation in experiments with *A. baylyi* suggest that this mechanism may play an important role in stress responses. BD4*_rev_* frequently evolved mutations in a universal stress protein in our evolution experiment. Two domesticated ADP1 strains in our reconstructed phylogeny had point mutations in the coding region of a separate universal stress protein. These findings suggest that modulating stress responses in ADP1 adapts it to laboratory conditions, although whether its stress responses have become more or less active cannot be determined from these mutations alone.

### Domestication impaired growth of *A. baylyi* strains on diverse carbon sources

Domesticated *A. baylyi* strains showed reduced growth or longer lag phases on glucose and the aromatic compounds benzoate and vanillate compared to BD4*_rev_*. Between the two extant groups of ADP1 strains, the more domesticated group, represented by ADP1(D) and ISx, grew worse on aromatic carbon sources than BD413*_rev_*. This could reflect reduction or loss of extraneous metabolic pathways or trade-offs due to mutations that are beneficial for growth on a limited range of carbon sources used in laboratory cultures. These pathways are of particular interest to research groups for their applications in metabolic engineering (15, 17, 44) and as a model system for studying molecular genetics and evolution (27, 44). Differences between strains could affect the reproducibility of these experiments as well as product yields. CsrA is classically involved in control of glycolysis and gluconeogenesis (41), and truncation of *csrA* slowed the growth of ADP1(D) on glucose relative to ISx. Truncation of *csrA* also impaired growth on vanillate, which suggests a role for CsrA in regulating aromatic catabolism in *A. baylyi*. Additionally, mutations around the vanillate metabolism and transport genes *vanA* and *vanK* and the *cat* genes likely also contributed to the reduced growth of ADP1(D) and ISx on aromatic compounds.

An additional unexpected finding was a novel 130 kb plasmid in the undomesticated strain ATCC 33304, not found in any of the domesticated strains. This plasmid was presumably present in the ancestor strain BD4 and could have been lost during UV mutagenesis or routine passaging over time. It encodes several antibiotic and heavy metal resistance transporters, as well as metabolic enzymes for the catabolism of plant-derived compounds, suggesting that it had a role in bacterial growth and survival in the soil environment from which BD4 was originally isolated. Domesticated *A. baylyi* strains also evolved mutations in chromosomal copies of two genes with homologs on this plasmid: *hpt* (hypoxanthine phosphoribosyltransferase) and *vanK* (vanillate transporter). These mutations may have evolved during domestication to compensate for the loss of the plasmid-encoded copies.

### Conclusion

We have shown that strains of ADP1 being used by different research groups have substantially different phenotypes. We recommend researchers sequence their *A. baylyi* strains to determine exactly which genotype they are working with and include that information in their publications to improve reproducibility. Our results also suggest avenues for rational strain improvement through precision genome editing of ADP1 that can recapitulate the benefits of mutations that evolved during domestication (e.g., elimination of cell chains and improved growth on common laboratory media) without their side effects and trade-offs (e.g., reduced competence and worse growth on glucose). Finally, reversion of mutations acquired during domestication may also improve the growth of *A. baylyi* strains on carbon sources such as aromatic compounds derived from lignin, an activity that is desirable for bioenergy and biomanufacturing applications.

##  MATERIALS AND METHODS

### Strains and culture conditions

Strains used in this study are described in Table 1. *A. baylyi* strains were grown at 30°C with shaking at 200 RPM in an incubator for liquid cultures in either LB medium or minimal media. LB medium contained per liter 10 g tryptone (VWR), 5 g yeast extract (Fisher), and 10 g NaCl (Fisher). When necessary for selection, 50 µg/mL kanamycin (Kan) (Sigma-Aldrich) or 200 µg/mL azidothymidine (AZT) (Sigma-Aldrich) was added to culture media.

The basal S2 medium of Juni and Janik was supplemented with different carbon sources (1). S2 medium contained per liter 0.75 g KH_2_PO_4_ (Sigma-Aldrich), 8.25 g Na_2_HPO_4_·2H_2_O (Sigma-Aldrich), 0.1 g MgSO_4_·7H_2_O (Sigma-Aldrich), 1.0 g NH_4_Cl (Fisher), 0.005 g CaCl_2_ (Fisher), 0.00025 g FeSO_4_·7H_2_O (Sigma-Aldrich), and 0.5 g glucose (Sigma-Aldrich). As described by Juni and Janik (1), this includes half the concentration of salts originally used in Monod’s S2 medium (45). The ferrous sulfate heptahydrate and carbon source were added by filter sterilization after autoclaving. For growth curves, carbon sources were substituted for glucose as listed in Fig. 5. For growth assays and microscopy, ferric chloride (FeCl_3_·6H_2_O, Fisher, 0.006 g/l final concentration) was substituted for ferrous sulfate, as it substantially improved growth. Vanillate and benzoate stocks were prepared as described by Luo et al. (27).

ABMS medium (46) was used for adaptive laboratory evolution and was made by combining 25 mL 20× mineral solution and 25 mL 20× phosphate buffer, both sterilized by autoclaving, followed by the addition of 10 mL 1 M sodium succinate (Sigma-Aldrich), sterilized by filtration, for a final volume of 500 mL. The 20× phosphate buffer (1,000 mL) contained 136 g KH_2_PO_4_ (Sigma-Aldrich) and 265 g Na_2_HPO_4_·H_2_O (Sigma-Aldrich). The 20× mineral solution (1,000 mL) contained 20 g NH_4_Cl (Fisher), 11.6 g MgSO_4_·7H_2_O (Fisher), 2 g KNO_3_ (Sigma-Aldrich), 1.34 g CaCl_2_·2H_2_O (Sigma-Aldrich), 0.04 g (NH_4_)_6_Mo_7_O_24_·4H_2_O (Sigma-Aldrich), and 20 mL SL9 trace minerals solution. SL9 trace minerals solution (1,000 mL) contained per liter 12.8 g nitrilotriacetic acid (Sigma-Aldrich), 2 g FeSO_4_·7H_2_O (Sigma-Aldrich), 0.208 g CoCl_2_ (Sigma-Aldrich), 0.244 g MnCl_2_·4H_2_O (Sigma-Aldrich), 0.14 g ZnCl_2_ (Sigma-Aldrich), 0.072 g Na_2_MoO_4_·2H_2_O (Acros Organics), and 0.026 g NiCl_2_ (Sigma-Aldrich). One milliliter of a 10× solution consisting of 0.06 g H_3_BO_3_ (Sigma-Aldrich) and 0.02 g CuCl_2_·2H_2_O (Sigma-Aldrich) in 10 mL H_2_O was added to the SL9 trace minerals solution, the pH was adjusted to 6.5, and then it was sterilized by autoclaving before use.

### Genomic DNA extraction, sequencing, assembly, and analysis

For short-read sequencing on an Illumina instrument, genomic DNA was extracted with a PureLink Genomic DNA Kit (Invitrogen) and sequenced as reported previously (36). For long-read sequencing, high molecular weight DNA was extracted from 1 mL of overnight culture grown in LB with a Quick-DNA HMW MagBead Kit (Zymo Research). DNA extracts were barcoded with an Oxford Nanopore Rapid Barcoding Kit 24 V14 and sequenced on a MinION Mk1C sequencer. To assemble the reference genome of ATCC 33304 (*A. baylyi* BD4), long reads were assembled using Trycycler (47) following initial assembly of subsampled reads with Flye (48). The assembly was then polished with short reads using Polypolish (49). A final polish was performed by identifying mutations from the same short read sequences by running *breseq* (50) and applying called mutations to the reference sequence. The assembled genome was then annotated with Prokka (22). The Acinetobacter Plasmid Typing v3.0 database (24) was used to identify the rep type of the plasmid pBD4-1. The p*dif* module was identified as described by Blackwell and Hall (24, 25).

Whole-genome sequencing data from published studies of unmodified *A. baylyi* genomes (14, 26, 27) were downloaded from NCBI Sequence Read Archive for comparative analyses (accessions SRR15734327, SRR20766519, and SRR28120535). Mutations in each genome were identified by using *breseq* (50) to compare Illumina reads from each strain against the assembled genomes of ATCC 33304 and ADP1(D) (37). The maximum parsimony tree of *A. baylyi* strains, which minimizes the number of inferred mutations, was constructed manually.

### Microscopy

Cultures were imaged on a Zeiss Axiovert 200M fluorescence microscope in the UT Austin Microscope and Imaging Facility. Ten microliters of overnight culture were placed on a glass slide and covered with a coverslip, then imaged using the 63× objective and DIC contrast. Afterward, images were cropped and contrast was adjusted by linear scaling in Fiji (51).

### Mutant construction

*A. baylyi* mutants were constructed using Golden Transformation (13) with the primers listed in Table S2. Briefly, a *tdk*/*kan* selectable/counterselectable cassette was transformed following ligation to homologous flanks targeting the desired gene amplified by PCR. Cells with successful insertion into the chromosome were selected on plates containing kanamycin. Isolated colonies from these plates were then inoculated into cultures in LB-Kan medium and grown overnight. The intended mutation was then introduced by recombination with a rescue cassette containing the mutation and 1 kb homologous flanks on each end, followed by selection for loss of the *tdk* gene on LB-AZT plates. Insertion of the *tdk*/*kan* cassette into *csrA* was unsuccessful; therefore, for the construction of the *csrA* truncation mutant, the cassette was inserted immediately downstream of the gene instead. Mutant constructs were verified by Illumina sequencing and compared to the ADP1(D) reference genome with *breseq* as described above. When mutating the *pgi* or *galU* genes, isolated colonies were streaked onto an additional LB-Kan or LB-AZT plate and grown overnight to ensure isolation of pure clones.

### Competence assays

Competence was measured by transforming each strain with genomic DNA from an ISx derivative with a spectinomycin-resistance gene replacing gene *ACIAD2049*. There are no mutations in any of the strains near *ACIAD2049* that could affect the frequency of recombination. For measurements of overall competence, 500 ng of genomic DNA of the spectinomycin-resistant ISx strain was added to 35 µL of eight replicate overnight cultures of each strain in 500 µL of LB medium and incubated overnight. All replicates for each assay were transformed at the same time. The following day, each transformation was serially diluted in 10-fold increments to 10^−8^ in sterile saline, and 5 µL of the dilutions were spot plated on LB plates with and without spectinomycin and incubated overnight before counting colonies to calculate the transformation rates. Transformation frequencies were calculated by dividing the CFUs/mL determined from selective plates by the CFUs/mL determined from non-selective plates.

For measurements of competence during growth, overnight cultures of each strain were diluted 1:25 by adding 2 mL of culture to 48 mL of fresh medium in 250 mL flasks. At each time point (3 h for log phase and 24 h for stationary phase), 500 µL aliquots were transferred to separate 25 mL culture tubes. These were inoculated with 500 ng of transforming genomic DNA and incubated for 30 min before adding 50 ng DNase I. Digestion of remaining DNA was allowed to proceed for 10 min with continued incubation at 30°C before diluting each transformation and plating it on selective and non-selective plates as mentioned above. All replicates for each time point were transformed concurrently.

### Growth assays

Growth curves were performed in a Tecan Infinite 200 Pro plate reader. For each medium, cultures were first inoculated into 5 mL cultures in 25 mL glass tubes and incubated overnight. Five microliters of these cultures was transferred to 5 mL of fresh medium and incubated overnight again to precondition. Then, 2 µL of each overnight culture was inoculated into 198 µL of medium in each well of a 96-well clear flat-bottom microplate (Corning Incorporated, catalog number 3596). OD_600_ readings were measured using the plate reader every 10 minutes for 24 h for six biological replicates per strain per medium. Cultures were incubated at 30°C with orbital shaking at an amplitude of 3.5 mm for 7 min between measurements. All replicates for each growth medium were included on the same 96-well plate and assayed concurrently.

### Adaptive evolution of BD4*_rev_*

Eleven colonies of GFP-tagged BD4_rev_ (strain MRV001, *gfp* inserted in place of *ACIAD2049*) were inoculated into separate culture tubes containing ABMS medium (46). These cultures were allowed to grow for 24 h before being diluted 1,000× into fresh ABMS medium. The cultures were transferred 30 times, which corresponds to ~300 generations of evolution (36). At this point, 1 mL of each culture was frozen in 20% (vol/vol) glycerol. One endpoint clonal isolate was obtained from each evolved population by streaking it onto an LB agar plate, then restreaking an isolated colony on another LB agar plate. The final isolated clones were picked and grown in LB medium, and 1 mL of each resulting culture was frozen in 20% (vol/vol) glycerol. The genomes of these clones were sequenced as described above.

## Data Availability

Genome sequencing data are available from the NCBI Sequence Read Archive under accession no. PRJNA1256545.

## SUPPLEMENTAL MATERIAL

File S1 aem.00936-25-s0001.txt Reference genome of *A. baylyi* strain ATCC 33304 (BD4) in GenBank format.

Table S1 aem.00936-25-s0002.xlsx Mutations in domesticated *A. baylyi* strains and isolates from adaptive laboratory evolution identified by breseq.

Table S2 aem.00936-25-s0003.xlsx Primers used in this study.

ASM does not own the copyrights to Supplemental Material that may be linked to, or accessed through, an article. The authors have granted ASM a non-exclusive, world-wide license to publish the Supplemental Material files. Please contact the corresponding author directly for reuse.
